# Enhancing the Amyloid-β Anti-Aggregation Properties of Curcumin via Arene-Ruthenium(II) Derivatization

**DOI:** 10.3390/ijms23158710

**Published:** 2022-08-05

**Authors:** Massimiliano Cuccioloni, Valentina Cecarini, Laura Bonfili, Riccardo Pettinari, Alessia Tombesi, Noemi Pagliaricci, Laura Petetta, Mauro Angeletti, Anna Maria Eleuteri

**Affiliations:** 1 School of Biosciences and Veterinary Medicine, University of Camerino, Via Gentile III da Varano, 62032 Camerino, MC, Italy; 2 School of Pharmacy, University of Camerino, Via Sant’Agostino, 62032 Camerino, MC, Italy; 3 School of Science and Technology, University of Camerino, Via Sant’Agostino, 62032 Camerino, MC, Italy

**Keywords:** Alzheimer’s disease, amyloid-β, curcumin, organoruthenium derivative, anti-aggregating molecule

## Abstract

Alzheimer’s disease (AD) is a fatal neurodegenerative disorder associated with severe dementia, progressive cognitive decline, and irreversible memory loss. Although its etiopathogenesis is still unclear, the aggregation of amyloid-β (Aβ) peptides into supramolecular structures and their accumulation in the central nervous system play a critical role in the onset and progression of the disease. On such a premise, the inhibition of the early stages of Aβ aggregation is a potential prevention strategy for the treatment of AD. Since several natural occurring compounds, as well as metal-based molecules, showed promising inhibitory activities toward Aβ aggregation, we herein characterized the interaction of an organoruthenium derivative of curcumin with Aβ(1–40) and Aβ(1–42) peptides, and we evaluated its ability to inhibit the oligomerization/fibrillogenesis processes by combining in silico and in vitro methods. In general, besides being less toxic to neuronal cells, the derivative preserved the amyloid binding ability of the parent compound in terms of equilibrium dissociation constants but (most notably) was more effective both in retarding the formation and limiting the size of amyloid aggregates by virtue of a higher hindering effect on the amyloid–amyloid elongation surface. Additionally, the complex protected neuronal cells from amyloid toxicity.

## 1. Introduction

More than 50 million individuals worldwide suffer from Alzheimer’s disease (AD), the most common form of fatal neurodegenerative dementia [1,2,3,4] that also cross-correlates with other diseases [5]. The expectation of life at diagnosis is 3–9 years [6], and aging is a major risk factor for AD, as the incidence of the disease increases exponentially every 5 years after 65 years of age [7].

Histologically, the accumulation of misfolded proteins as plaques in the aging brain is among the main hallmarks of AD: this triggering event causes oxidative and inflammatory damage, which, in turn, leads to energy failure and synaptic dysfunction [8]. These plaques are mainly constituted by the amyloid-β (Aβ) peptide [9], with the two main alloforms being 42 and 40 amino acids long (Aβ(1–42) and Aβ(1–40), respectively) [10,11]. These peptides derive from the amyloid precursor protein through sequential proteolysis by the aspartyl protease β-secretase and presenilin-dependent γ-secretase cleavage [12]. Despite its lower abundancy in the brain, Aβ(1–42) has a higher tendency to aggregate and is more neurotoxic [13,14,15,16]. According to the amyloid cascade hypothesis, the increased production and accumulation of the Aβ peptide promote the formation of Aβ oligomers, protofibrils, and, ultimately, amyloid fibrils that lead to neurodegeneration [17,18], with cholesterol [19] and other lipids [20] promoting the aggregation process. Although initial studies on Aβ were mainly focused on the fibrillar deposits of the peptide, the earlier phases of Aβ assembly, involving the formation of oligomers, recently attracted the attention of clinicians and researchers since these aggregates were demonstrated to be far more toxic than fibrils to neurons [21]. Several molecular aspects of the “amyloid cascade” were dissected in the last few years, inspiring the development of novel types of AD therapeutics. Among these, naturally occurring small-molecule inhibitors of amyloid aggregation, including monomeric polyphenols [22,23,24,25] and organometallic complexes [26,27,28], displayed promising results. In particular, metal-based compounds showed remarkable biological and pharmacological properties and have long been used as chemotherapy agents [29]. In the last decade, ruthenium-based complexes emerged as valuable second-generation metallodrugs since they were associated with several significant advantages, including higher tolerability and selectivity than Pt(II) [30], as well as therapeutic efficiency with respect to other metal ions such as Zn(II), Cu(II), and Fe(III), which were also demonstrated to promote Aβ aggregation under physiological conditions [31]. To further reduce the metal-associated toxicity, naturally occurring compounds, such as polyphenols, have been rationally combined to metals, as they can form stable coordination complexes. In turn, polyphenols themselves can benefit from complexation since metals can increase their bioavailability and tune their biological properties [32,33,34,35]. Among the most exploited polyphenols, curcumin has gained enormous attention for its several biological and therapeutic properties [36,37], with experimental evidence demonstrating its neuroprotective role in vitro [38] and in AD models [39], the possibility to enhance its anticancer and antimicrobial activities, as well as further expand its multi-target nature via Ru(II)-complexation [32,40,41].

Based on these evidences, in this study, we explored the amyloid binding and anti-aggregating abilities of a bioactive half-sandwich organo-ruthenium(II) complex of curcumin [32] by integrating in silico and in vitro approaches. Interestingly, the derivative showed higher efficacy than parent curcumin in blocking amyloid-β aggregation, as oligomers and fibrils, at the same time, are protecting neuronal cells from amyloid toxicity.

## 2. Results and Discussion

### 2.1. Molecular Docking

The binding modes of curcumin and RuCurcumin with Aβ(1–40) or Aβ(1–42) monomers were rationalized by molecular docking studies. In terms of binding affinity, p-cym-Ru modification induced a low-to-negligible effect: in fact, irrespective of the different energy contributions to the stabilization of each complex (Aβ-curcumin complexes were characterized by higher electrostatic and lower Van der Waals contributions, respectively, compared with the derivative counterparts), comparable values of binding affinities in the low micromolar range were computed (Table 1). In addition, and consistently with the higher hydrophobic nature of the Aβ(1–42) peptide, the complexes thereof with curcumin/RuCurcumin presented a dominant VdW contribution to the interaction energy.

**Figure 1:**
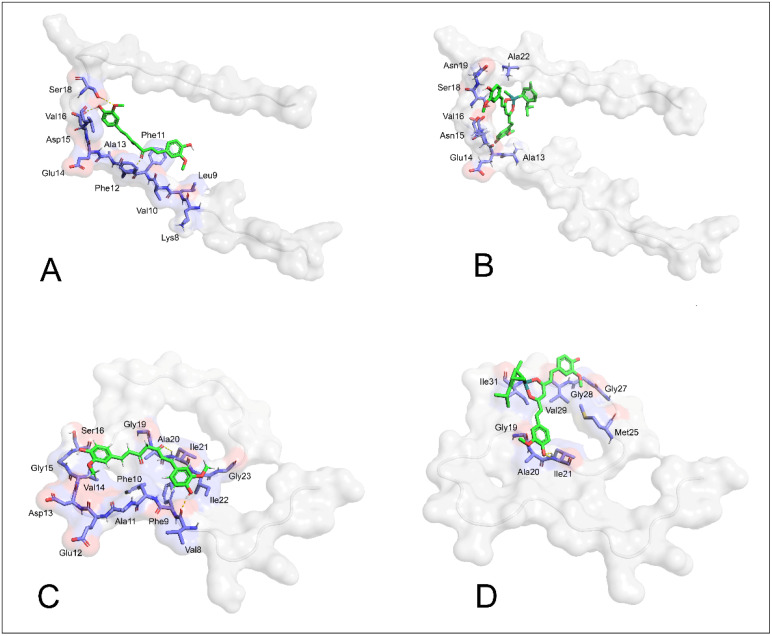
Characterization of the binding interface of the protein−ligand complexes based on molecular docking. Comparative visualization of binding models of curcumin and RuCurcumin with Aβ(1–40) (insets (**A**,**B**)) and Aβ(1–42) monomers (insets (**C**,**D**)). Amyloid-β peptides are displayed as transparent grey surfaces, and residues constituting the binding regions are rendered as light blue sticks.

Structurally, the beta-turn region of the U-shaped amyloid 1–40 monomer was calculated to be the most likely to accommodate the molecules of interest. Specifically, parent curcumin and its Ru-derivative targeted two largely overlapping regions of the amyloid 1–40 monomer, with amino acids Ala13, Glu14, Asp15, Val16, and Ser18 being common to both binding interfaces (Figure 1, Panels A and B).

Differently, parent curcumin and its Ru-derivative were calculated to target two contiguous, but distinct, regions of the 1–42 amyloid monomer (Figure 1, Panels C and D). Both for amyloid 1–40 and 1–42, only curcumin was predicted to form H-bonds with the amyloid proteins, proving the importance of the availability of the beta dicarbonyl group in establishing polar interactions. Globally, the p-cym-Ru moiety protected curcumin from deformations in its planarity and altered the interaction angle of the molecule to the plane of the amyloid (the elongation surface [42]), but it did not participate in any interaction with the protein. Nevertheless, based on these models and on crystallographic data available for the 1–40 and 1–42 oligomers, according to which oligomers consist of monomers that are stacked atop each other [43,44], the calculated binding modes of curcumin and its Ru-derivative (in particular) were likely to hinder the self-assembly process of amyloid peptides that leads to oligomerization.

### 2.2. Biosensor Binding Study

First, the amyloid binding ability of curcumin and RuCurcumin was assessed and compared using a general biosensor-based approach, based on the interaction between a surface-blocked protein and the soluble molecules of interest [45].

Both molecules interacted with Aβ(1–40) and Aβ(1–42) monomer peptides, specifically and reversibly (Figure S1), with moderate affinities (equilibrium dissociation constants were in the low-to-sub micromolar range—Table 2). The modest negative effect on the binding affinity, due to the chemical modification of curcumin, was mainly attributed to the slow recognition phase between RuCurcumin and both amyloid peptides (lower values of k_ass_), in line with the computed lower electrostatic energy contribution.

Based on these results and on the computational study, according to which both curcumin and RuCurcumin could interact with amyloid peptides and (potentially) antagonize the amyloid–amyloid dimerization, we performed a competitive binding assay that exploited the same amyloid-β functionalized surfaces.

**Figure 2:**
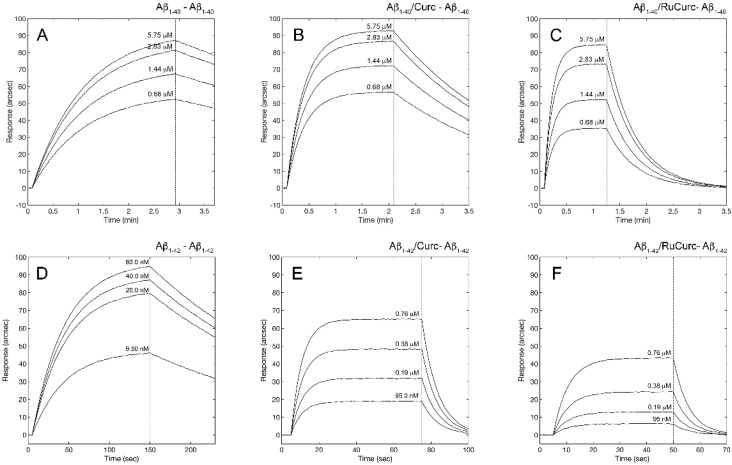
Biosensor binding study. Effect of curcumin and RuCurcumin on the kinetics of Aβ(1–40) and Aβ(1–42) dimerization.

Interestingly, the pre-saturation of independent surface-blocked amyloid peptides with 20 μM curcumin or RuCurcumin, significantly interfered with the formation of amyloid–amyloid complexes, as evident from the general increase in the values of dissociation kinetic and equilibrium parameters (Table 3) and (most evidently in the case of Aβ(1–42)) from the reduction in the maximal responses at equilibrium (Figure 2), which indicates the formation of lower molecular weight amyloid aggregates.

Globally, RuCurcumin showed the highest destabilizing effect on amyloid 1-42 dimers, with a nearly 50-fold decrease in binding affinity.

### 2.3. Effects on Amyloid Peptide Aggregation

**Figure 3:**
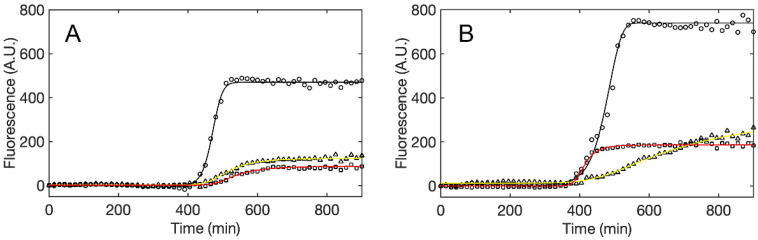
Comparison of aggregation kinetics of Aβ(1–40) and Aβ(1–42) fibrils ((**A**) and (**B**) panels, respectively) grown at 37 °C, in the absence (○) and in the presence of 20 µM curcumin (△) and RuCurcumin (□), without continuous agitation.

To test the effective inhibitory ability of curcumin and RuCurcumin against amyloid-β aggregation, we monitored kinetics of fibril formation using a thioflavin T (ThT) fluorescence assay. Our data proved that the aggregation into fibrils of Aβ(1–40) and Aβ(1–42) peptides was impaired in the presence of 20 µM curcumin/RuCurcumin, with respect to ligand-free control (Figure 3).

The two molecules showed a similar anti-aggregation activity against Aβ(1–40), with the Ru-derivative being more effective in limiting fibril elongation. Conversely, more significant differences occurred with Aβ(1–42). Specifically, RuCurcumin had a minor effect on the Aβ(1–42) nucleation, as evident from the negligible shift in the point of transition with respect to ligand-free aggregation (*c* parameter, Table S2), but it effectively limited the maximum elongation of the fibril, with a significantly lower value and lower rate of approach to the second plateau, with respect to ligand-free control (*d* and *e* parameters, respectively, Table S2). On the other end, curcumin exerted an initial anti-aggregation effect that significantly retarded the nucleation phase of Aβ(1–42) amyloid more efficiently than RuCurcumin (this result was consistent with the faster formation of the Aβ(1–42)-curcumin adduct, as shown from the biosensor binding analysis). Nevertheless, the increasing trend toward a higher value of saturation demonstrated that curcumin was less effective than its Ru-derivative in limiting the maximum elongation of the fibril (Table S1). In this case, no reliable quantitative comparison in kinetic parameters was possible since ThT fluorescence data, in the presence of curcumin, did not reach saturation in the timeframe considered.

### 2.4. Immunoblot of Curcumin- and RuCurcumin-Treated Amyloid Oligomers

**Figure 4:**
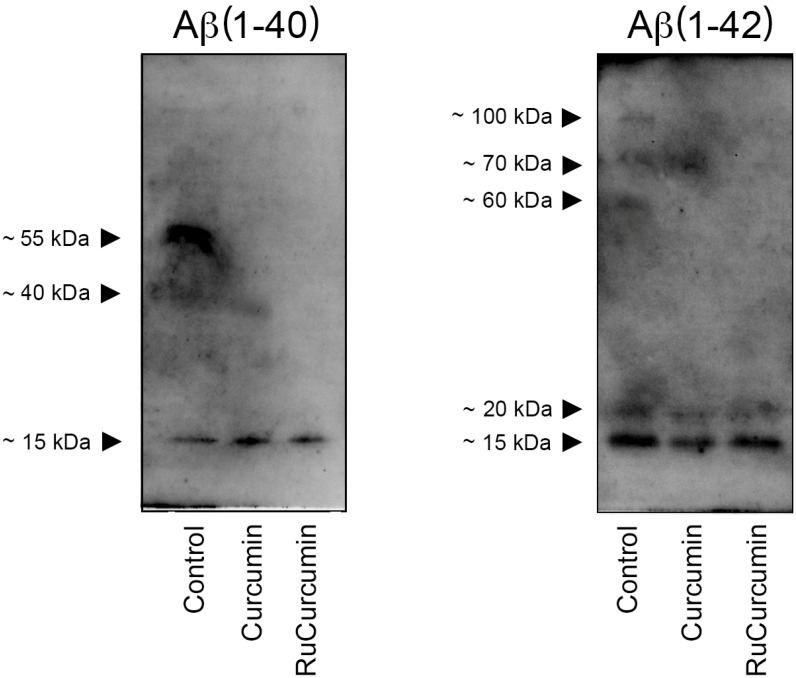
Western blot of Aβ(1–40) and Aβ(1–42) oligomers grown for 24 h, under the conditions described in the Materials and Methods section, in the presence and in the absence of 20 µM curcumin or RuCurcumin.

Chemically modified curcumin showed enhanced anti-oligomerization properties as well. Aβ(1–40) aggregates, grown in the absence of any co-treatment, showed three bands approximately at 55 kDa, 40 kDa (corresponding to medium-size oligomers) and at 15 kDa (corresponding to tetramer). The band at 55 kDa was not detectable upon treatment with curcumin, whereas the effects of the treatment with RuCurcumin were even more evident, with only the 15 kDa band being still observed (Figure 4, Panel A). Similarly, Aβ(1–42) aggregates, grown in the absence of any co-treatment, showed two bands approximately at 15 and 20 kDa, corresponding to tetra and pentamers, and three bands at 60–100 kDa, corresponding to higher size oligomers (Figure 4, Panel B—Control lane). The highest molecular weight band disappeared in the aggregates grown in the presence of curcumin, with a smeared band at approx. 70 kDa and the lower MW bands still being present (Curcumin lane). Most interestingly, the treatment with RuCurcumin successfully interfered with the formation of high molecular weight oligomers, with only the low molecular weight bands associated with tetra and pentamers still being detectable (RuCurcumin lane).

### 2.5. SEM

**Figure 5:**
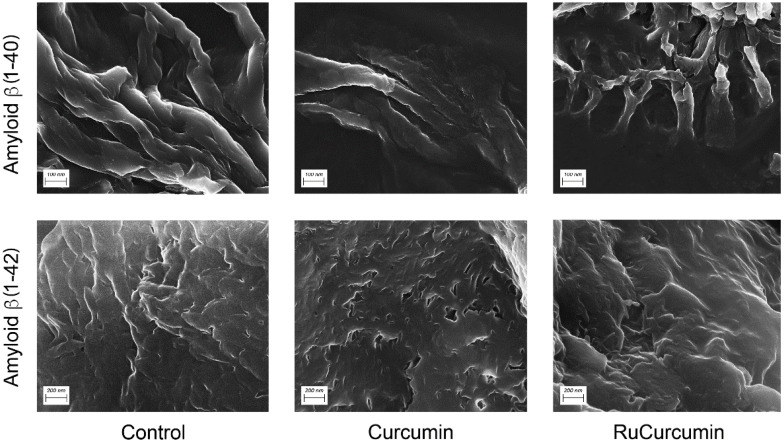
SEM analysis of amyloid aggregates. Comparison of the morphology of Aβ(1–40) and Aβ(1–42) fibril aggregates in the absence and in the presence of 20 µM curcumin and RuCurcumin.

To obtain information on the morphology of Aβ fibril aggregates, scanning electron microscopy analyses were performed on stub-deposited amyloid aggregates grown in the presence and in the absence of 20 µM curcumin/RuCurcumin, as described in the Materials and Methods. Morphologically, large β-amyloid plaque deposits (with a mean diameter in the range 50–80 μm) were generally observed. These deposits consisted of Aβ fibril network structures with peculiar arrangements. Amyloid 1-40 and 1-42 plaques showed major differences in terms of structural organization (Figure 5). Specifically, Aβ(1–40) amyloid deposits were characterized by individual fibrils wrapped around one another (Figure 5, Upper panels), whereas Aβ(1–42) amyloid deposits consisted of a thick network of amyloid fibrils (Figure 5, Lower panels). Most notably, individual treatments with curcumin and RuCurcumin caused a decrease in both amyloid 1-40 and 1-42 network density, with the Ru-derivative being more effective than curcumin. In addition, a general decrease in the diameter of fibril structures (Table S2) was observed upon treatment in Aβ(1–42) plaques more evidently than in Aβ(1–40) plaques, with RuCurcumin always being more effective than the parent curcumin.

### 2.6. Cell Membrane Permeability

**Figure 6:**
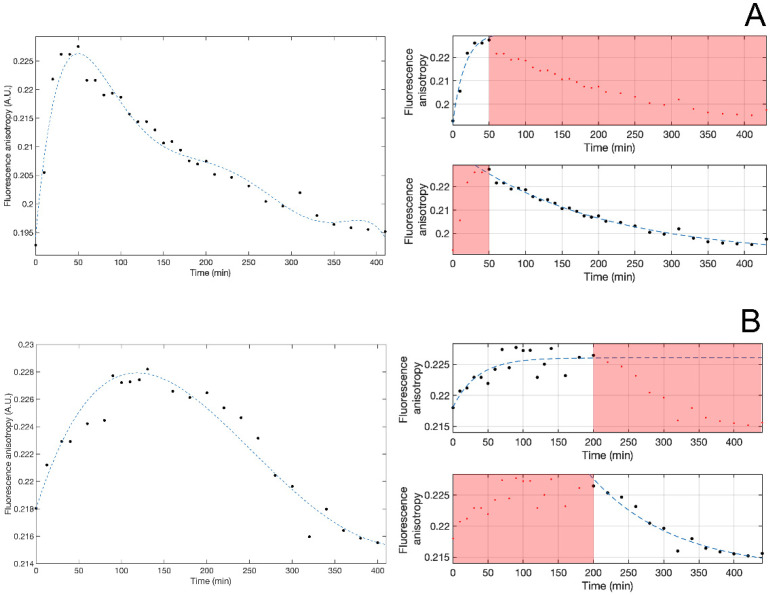
Visualization of changes in emission anisotropy, with time observed upon the SH-SY5Y cell membrane passage of curcumin (Panel (**A**)) and RuCurcumin (Panel (**B**)). Kinetic analysis of entry and release stages (right insets).

The passage of curcumin and RuCurcumin, across the membrane of SH-SY5Y neuronal cells, was compared by monitoring the changes in membrane fluidity using a trimethylammonium diphenylhexa-triene (TMA-DPH) fluorescent probe, as previously reported [33]. Upon curcumin treatment, SH-SY5Y cells labelled with TMA-DPH showed a rapid increase in emission anisotropy, peaking at 50 min (initial membrane insertion), followed by a slow decay (progressive cellular internalization), resulting in baseline recovery at 450 min (Figure 6, left panels). Although showing a general similar trend, kinetic analysis of raw data (summarized in Table S3) revealed that RuCurcumin displayed a less rapid membrane insertion than parent curcumin (RuCurcumin presented a lower value of k_in_) and longer permanence in the membrane, which is most likely due to the higher structural complexity. Conversely, curcumin was not significantly retained in the membrane. Irrespective of these initial differences, the internalization process globally occurred in a fully comparable timeframe for both molecules (400 min), given the faster release rate from the membrane of RuCurcumin (Figure 6, right panels).

### 2.7. Effect on Amyloid Citotoxicity

**Figure 7:**
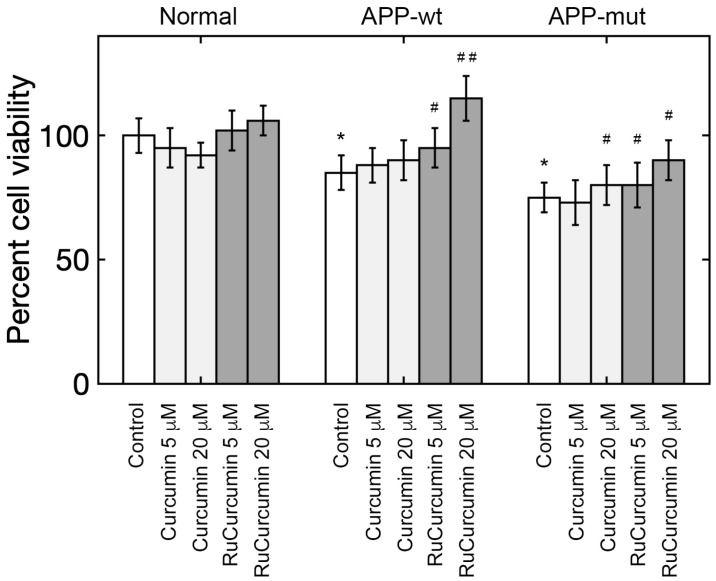
Effect of different concentrations of curcumin and RuCurcumin on normal and APP-transfected SH-SY5Y cells’ viability. Data points marked with an asterisk or hashtag are statistically significant with respect to normal control and to internal controls (APP-wt and APP-mut), respectively, to internal respective controls (*/^#^*p* < 0.05; ^##^ *p* < 0.01).

To evaluate the protective effects of RuCurcumin on a neuronal cell model, SH-SY5Y cells (normal and stably transfected, either with wild type or mutated amyloid precursor protein (APPwt and APPmut, respectively), were treated with subtoxic concentrations of RuCurcumin for 24 h. Comparative analyses revealed that the higher amyloid production (normal > APPwt > APPmut) [46] was associated with the progressively higher toxicity; most notably, RuCurcumin (more effectively than parent curcumin, Figure 7), protected neuronal cells against Aβ-induced cell death.

## 3. Materials and Methods

### 3.1. Synthesis of [Ru(cym)(curc)Cl]

**Scheme 1:**
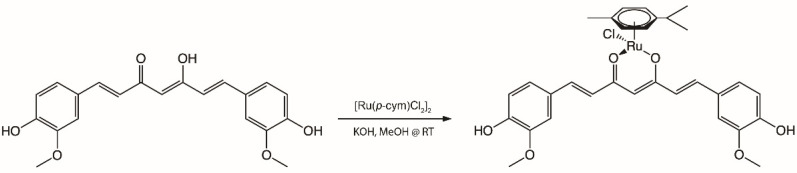
Synthetic procedure of p-cym-Ru(II)-curcumin.

The compound [Ru(cym)(curc)Cl] was prepared by modifying the previously described procedure [34] (Scheme 1).

The molecular structure of the complex was also previously reported and discussed [40]. Curcumin (186.4 mg, 0.504 mmol) was dissolved in methanol (20 mL) and KOH (28.4 mg, 0.504 mmol)) was added. The mixture was stirred for 1 h at room temperature and then [(p-cym)RuCl_2_]_2_ (0.154 g, 0.252 mmol) was added. The mixture was stirred for 24 h at room temperature and an orange precipitate formed, which was removed by filtration and washed with n-hexane (128 mg, 0.20 mmol, yield 80%). The residue was concentrated to 2 mL and stored at 4 °C. Red crystals formed over several days. The compound is soluble in acetone, acetonitrile, chlorinated solvents, DMF, and DMSO. Λ_m_ (CH_3_OH, 298 K, 10^−3^ mol/L): 22 S cm^2^ mol^−1^. Λ_m_ ((CH_3_)_2_SO, 298 K, 10^−3^ mol/L): 2 S cm^2^ mol^−1^. IR (nujol, cm^−1^): 3220 m br ν(OH), 1619 m, 1591s, 1500vs ν(C=O, C=C); 1H NMR (CDCl_3_, 293K): δ, 1.39 (d, 6H, CH(CH_3_)_2_ of p-cym), 2.34 (s, 3H, CH_3_ of p-cym), 2.98 (m, 1H, CH(CH_3_)_2_ of p-cym), 3.93 (s, 6H, OCH_3_ of curc), 5.45 (s, 1H, C(1)H of curc), 5.55br, 5.84br (4H, AA′BB′ system, CH_3_-C_6_H_4_-CH(CH_3_)_2_ of p-cym), 6.42 (d, 2H, C(3, 3′)H of curc, ^3^J_trans_ = 15 Hz), 6.91 (br, 2H, C(9, 9′)H of curc), 7.00 (br, 4H, C(10, 10′)H and C(6, 6′)H of curc), 7.52 (d, 2H, C(4, 4′)H of curc, ^3^J_trans_ = 16 Hz); ^13^C [33] NMR (CDCl_3_, 293K): δ, 18.3 (s, CH_3_-C_6_H_4_-CH(CH_3_)_2_), 22.7 (s, CH_3_-C_6_H_4_-CH(CH_3_)_2_), 31.1 (s, CH_3_-C_6_H_4_-CH(CH_3_)_2_), 56.1 (s, O-CH_3_ of curc), 79.3, 83.2, 97.8, 99.8 (s, CH_3_-C_6_H_4_-CH(CH_3_)_2_), 102.1 (s, C(1, 1′) of curc), 109.3 (s, C(6, 6′) of curc), 114.9 (s, C(9, 9′) of curc), 122.7 (s, C(10, 10′) of curc), 125.6 (s, C(5, 5′) of curc), 128.7 (s, C(3, 3′) of curc), 138.9 (s, C(4, 4′) of curc), 146.9 (s, C(7, 7′) of curc), 147.3 (s, C(8, 8′) of curc), 178.5 (s, C(2, 2′)=O of curc). ESI-MS (+) CH_3_OH (m/z, relative intensity %): 603 [100] [(p-cym)Ru(curc)]^+^. IR spectra were recorded from 4000 to 30 cm^−1^ on a PerkinElmer Frontier FT-IR instrument. Additionally, ^1^H and ^13^C NMR spectra were recorded with a 500 Bruker Ascend^TM^ (500 MHz for ^1^H, 125 MHz for ^13^C) and a 400 Mercury Plus Varian (400 MHz for ^1^H, 100 MHz for ^13^C) instrument operating at room temperature, relative to TMS. {^1^H,^13^C}-HSQC and {^1^H,^13^C}-HMBC NMR spectra were recorded with a 500 Bruker Ascend^TM^ (500 MHz for ^1^H, 125 MHz for ^13^C) operating at room temperature, relative to TMS. Positive and negative ion electrospray ionization mass spectra (ESI-MS) were obtained on a Series 1100 MSI detector HP spectrometer using methanol as the mobile phase. Solutions (3 mg/mL) were prepared using reagent-grade methanol. Masses and intensities were compared to those calculated using IsoPro Isotopic Abundance Simulator, version 2.1.28. Melting points are uncorrected and were recorded on a STMP3 Stuart scientific instrument and on a capillary apparatus. Samples for microanalysis were vacuum-dried to a constant weight (20 °C, ca. 0.1 Torr) and analysed on a Fisons Instruments 1108 CHNS-O elemental analyser.

### 3.2. Molecular Docking

The molecular models of the complexes between Aβ(1–40) and Aβ(1–42), and the molecules of interest were obtained according to flexible ligand-receptor docking using Autodock 4.2 [47]. Ruthenium atom parameters used were “atom par Ru 2.96 0.056 12.000 -0.00110 0.0 0.0 0 -1 -1 1 # Non H-bonding” [48]. The 3D structures of curcumin (Pubchem ID: 969516) and RuCurcumin [32] were docked onto amyloid monomers extracted from the crystallographic structures of Aβ(1–40) (PDB ID: 2M4J [43]) and Aβ(1–42) (PDB ID: 2MXU, [44]), which were retrieved from the RCBS Protein Data Bank [49]. Specifically, a grid box (20 × 30 × 30 Å) was individually placed around the amyloid monomers, covering the entire surface, and extending 10, 15, and 15 Å in each direction. Unless stated differently, default settings were used throughout. The resulting models were analysed with Maestro (Schrödinger Release 2021-2: Maestro, Schrödinger, LLC, New York, NY, USA, 2021) and PyMOL (The PyMOL Molecular Graphics System, Version 2.4 Schrödinger, LLC).

### 3.3. Binding Study

Commercial Aβ(1–40) and Aβ(1–42) were dissolved to 1 mM in HFIP and immediately aliquoted in sterile tubes; the solvent was removed, under vacuum, at −20 °C, and the resulting peptide films were stored at −80 °C until use [50]. Binding experiments were performed on a resonant mirror biosensor (IAsys plus—Affinity Sensors Ltd., Cambridge, UK), equipped with carboxylate cuvettes (NeoSensors, Ltd., Manchester, UK). Independent surfaces were functionalized with Aβ(1–40) and Aβ(1–42), using a general protocol for proteins’ immobilization [45]. In brief, carboxylate surfaces were extensively rinsed and equilibrated with PBS pH 7.4, prior to the carboxylic group’s activation by EDC/NHS chemistry [51]. Amyloid films were dissolved in DMSO and diluted in 10 mM CH_3_COONa, pH 4.5 (pH value being chosen based on peptides’ isoelectric point), then covalently coupled to independent carboxylic surfaces via the N-terminus of Lys residues. Different stock solutions of Aβ(1–4x), with concentrations in the range 50–400 μg/mL, were tested to achieve optimal surface density: 100 μg/mL was finally selected, as it minimized steric hindrance and, at the same time, prevented the dimerization between blocked peptides, with both events being likely to reduce the number of available binding sites on the sensing surface. Free carboxylic sites on the sensor surface were inactivated with 1 M ethanolamine, pH 8.5, before surface re-equilibration with PBS. The resulting shifts in the baseline (ΔR = 360 arcsec) generally indicated the assembly of a partial monolayer for a 4 kDa protein (approximately 80% surface occupancy), corresponding to a final surface density of 0.60–0.70 ng/mm^2^, equivalent to 4.5–5 mg/mL. Negative baseline drift signals in Aβ surfaces were not observed with time or upon multiple washes, confirming that the amyloid oligopeptides were irreversibly anchored to the sensor surface. The temperature of the sensing chamber was set at 37 °C throughout. Next, immobilized Aβ peptides were added with different concentrations of the ligands of interest (either Aβ peptides, curcumin or RuCurcumin), each time monitoring association kinetics up to the plateau, and assessing baseline recovery among consecutive additions.

### 3.4. In Vitro Aggregation of Aβ Peptides

ThT fluorescence assay was used to quantitatively monitor the role of curcumin and RuCurcumin on the aggregation kinetics of amyloid fibrils. Aβ(1–40) and Aβ(1–42) films were re-dissolved to a final concentration of 100 μM in 10 mM HCl and incubated, at 37 °C, for 24 h to promote the formation fibrils [50]. Aggregation rates were monitored in a 96-well sealed plate, each well containing 10 μM Aβ (either 1-40 or 1-42), 40 μM ThT in the presence or in the absence of curcumin or RuCurcumin at 20 μM, in a total volume of 100 μL. The plate was analysed in a SpectraMax Gemini XPS microplate reader (Molecular Device, Milan, Italy) set at 4 °C for oligomerization and at 37 °C for fibrillogenesis. ThT fluorescence was recorded at 15 min intervals for 12 h (λ_exc_ = 440 nm, λ_em_ = 485 nm). The plate was shaken for 10 s before each reading. Background ThT fluorescence was subtracted from all readings. Aggregation kinetic parameters were obtained by fitting fluorescence raw data points to the sigmoidal curve in the following five-parameter equation [52] using MATLAB:

(1) $$F=d+\frac{\left(a-d\right)}{{\left(1+{\left(\frac{t}{c}\right)}^{b}\right)}^{e}}$$

where *t* is time, a and d control the position of the first and the second horizontal asymptotes, respectively, *b* controls the slope of the transition between asymptotes and together with *e* control the rate of approach to the second asymptote, and *c* controls the position of the transition region.

### 3.5. Immunometric Assay

Aβ(1–40) and Aβ(1–42) films were re-dissolved to a final concentration of 100 μM in F12 cell media and incubated at 4 °C, for 24 h, to promote the formation of oligomers [50]. Oligomers of amyloid peptides were produced in the presence and in the absence of 20 μM curcumin or RuCurcumin, separated by SDS-PAGE using NuPAGE^®^ Novex 12% Bis–Tris Gel, and electroblotted onto PVDF membranes. Membranes with transferred proteins were blocked overnight at 4 °C in TTBS (Tween-20, 10 mM Tris-HCl, and 0.5 M NaCl), containing 5% bovine serum albumin, and then incubated with an anti-oligomer A11 polyclonal antibody. The immunoblot detection was performed with an ECL Western blotting analysis system (Biorad, Hercules, CA, USA). Each gel was loaded with molecular mass markers in the range of 20–245 kDa (Prestained Protein MW markers, Euroclone, Milan, Italy).

### 3.6. SEM Analysis

Aβ(1–40) and Aβ(1–42) peptide films were re-suspended in 10 mM HCl, to a final concentration of 10 μM, and incubated at 37 °C, for 24 h, in the presence and in the absence of 20 μM curcumin or RuCurcumin. Each resulting solution (100 μL) was loaded onto individual SEM stubs and dried by stepwise ethanol treatment to preserve the nanostructures of β-amyloid fibrils [53]. Resulting protein deposits were coated with chromium, using a Q150T ES metallizer (Quorum Technologies Ltd., East Sussex, UK). The morphology of differently assembled fibril plaques was evaluated on a Sigma 300, Zeiss, Gina, Germany operating at 7 kV, equipped with an energy dispersive X-ray spectroscopy (EDX, Quantax, EDS, Bruker, Billerica, MA, USA).

### 3.7. Cell Membrane Permeability

TMA-DPH probe (λ_exc_ = 340 nm; λ_em_ = 460 nm) was used to monitor compound cell internalization by following the changes in membrane fluidity [54] of SH-SY5Y cells independently treated with either 20 μM curcumin and RuCurcumin. In detail, 1.5 × 10^5^ SH-SY5Y cells per mL were incubated with 1 μM TMA-DPH, then individually added with the compounds. Fluorescence anisotropy (r) was measured for 400 min, at 10-min intervals, and was calculated as previously reported [33]. Anisotropy measurements were carried out in an RF-5301PC Shimadzu spectrofluorometer thermostatted at 37 °C. The kinetic rate constants characterizing the main steps of the permeation event (namely, *k_in_* and *k_out_*) were derived according to a general mono-exponential model:

$$r_{in}=a\left(1-e^{k_{in}t}\right)+c$$

$$r_{out}=b\left(e^{k_{0ut}t}\right)+d$$

where *r_in_* and *r_out_* are the fluorescence anisotropy intervals corresponding to drug entry and exit phases from the membrane, respectively.

### 3.8. Cell Viability

SH-SY5Y cells were cultured in 1:1 Dulbecco’s modified Eagle’s medium and Nutrient Mixture F12 containing 10% foetal bovine serum (FBS), 2 mM glutamine, 100 units mL^−1^ penicillin, and 100 μg mL^−1^ streptomycin at 37 °C in a 5% CO_2_-containing atmosphere. The SH-SY5Y cells’ stable transfection with wild type AβPP 751 (APPwt) and AβPP (Val717Gly) mutations (APPmut) was performed as described elsewhere [55]. These cells were a kind gift of Prof. Daniela Uberti from the University of Brescia. Stably transfected cells, expressing either the APPwt or the APPmut construct, were maintained in the SH-SY5Y medium added with G418 at a final concentration of 600 μg mL^−1^. Cell viability was evaluated with the 3-(4,5-dimethylthiazol-2-yl)-2,5-diphenyltetrazolium bromide assay (MTT) [56]. Upon 24 h treatment with increasing concentrations (0–150 μM) of curcumin and RuCurcumin dissolved in DMSO, cells were washed in PBS, pH 7.5, and then, MTT (final concentration 0.5 mg mL^−1^) was added to the culture medium without FBS and incubated, for 2 h, at 37 °C. The medium was then removed and replaced with 100 μL of DMSO. The optical density was measured at 550 nm in a microtiter plate reader. At least six cultures were utilized for each time point.

### 3.9. Statistical Analysis

Results presented in this study are expressed as mean values, with their standard deviations obtained from tree separate experiments. Statistical analysis was performed with one-way ANOVA, followed by the Bonferroni test using MATLAB R2021b. *p*-values < 0.05 and < 0.01 were considered significant.

## 4. Conclusions

AD is a multifactorial neuropathology related to several biological and physiological alterations. Currently available drugs can ameliorate cognitive, behavioural, and functional deficits, but they cannot stop the progression of AD.

Herein, we tested an arene Ru(II) curcumin complex for its anti-aggregating properties toward amyloid 1-40 and 1-42 peptides. The modified curcumin was shown to reversibly bind both peptides with moderate affinity and interfere with the stacking mechanisms of the aggregation. Most interestingly, experimental evidence and molecular modelling data demonstrated that the chemical modification of curcumin improved its efficacy, most likely due to a higher hindering effect on the amyloid aggregation mechanism.

These results supported the hypothesis that the biological properties of naturally occurring compounds can be properly tuned upon chemical modification and, case in point, that curcumin derivative can prevent amyloid aggregation and, consequently, reduce the cellular toxicity of Aβ aggregates. Globally, our data may pave the way for the exploration of more potent amyloid inhibitors (with low toxicity toward neuronal cells), which are also able to disrupt existing aggregates, based on the rational modification of naturally occurring molecules.

## Figures and Tables

**Table 1:** Computed affinities and energy contribution (kcal/mol) values for the complexes formed between the molecules of interest and amyloid 1–40 and 1–42 monomers.

	K_D_ (µM)	ΔG	Total Energy	Internal Energy	VdW Energy	Elect. Energy
**Curcumin-Aβ(1–40)**	1.24	−7.343	17.421	−49.234	−8.350	−40.884
**RuCurcumin-Aβ(1–40)**	1.25	−7.411	12.232	−38.787	−8.927	−29.860
**Curcumin-Aβ(1–42)**	0.96	−8.208	36.409	−27.180	−15.957	−11.223
**RuCurcumin-Aβ(1–42)**	2.02	−7.764	34.203	−28.255	−19.452	−5.943

**Table 2:** Comparison of kinetic and equilibrium parameters for the binding of curcumin and RuCurcumin to amyloid peptides.

	K_D_ (µM)	*k_ass_* (M^−1^s^−1^)	*k_diss_* (s^−1^)
**Aβ(1–40)-RuCurcumin**	1.46 ± 0.12	1190.68 ± 52.20	(1.74 ± 0.12) × 10^−3^
**Aβ(1–40)-Curcumin**	0.82 ± 0.38	6265.85 ± 1810.06	(5.11 ± 1.87) × 10^−3^
**Aβ(1–42)-RuCurcumin**	1.60 ± 0.69	1973.84 ± 848.12	(3.15 ± 1.00) × 10^−3^
**Aβ(1–42)-Curcumin**	0.74 ± 0.29	4598.57 ± 876.21	(3.48 ± 1.00) × 10^−3^

**Table 3:** Competitive effect on amyloid-amyloid binding. Comparison of kinetic and equilibrium parameters for the amyloid-amyloid binding in the absence and in the presence of saturating curcumin (*) and RuCurcumin (**).

	K_D_ (nM)	*k_ass_* (M^−1^s^−1^)	*k_diss_* (s^−1^)
**Aβ(1–40)-Aβ(1–40)**	458 ± 91	1247 ± 58	0.0006 ± 0.0001
Aβ(1–40)/Curcumin *-Aβ(1–40)	441 ± 22	3900 ± 1400	0.0017 ± 0.0005
Aβ(1–40)/RuCurcumin **-Aβ(1–40)	1060 ± 150	7300 ± 1800	0.0078 ± 0.0007
**Aβ(1–42)-Aβ(1–42)**	6.48 ± 3.00	546,000 ± 112,000	0.0035 ± 0.0017
Aβ(1–42)/Curcumin *-Aβ(1–42)	212 ± 122	61,400 ± 29,600	0.029 ± 0.010
Aβ(1–42)/RuCurcumin **-Aβ(1–42)	322 ± 55.7	142,000 ± 21,800	0.044 ± 0.005

## Data Availability

Not applicable.

## Supplementary Materials

The following supporting information can be downloaded at: https://www.mdpi.com/article/10.3390/ijms23158710/s1.
