# Supplementary material for: Enhancing the Amyloid-β Anti-Aggregation Properties of Curcumin via Arene-Ruthenium(II) Derivatization
Source: Int J Mol Sci. 2022 Aug 5;23(15):8710. doi: 10.3390/ijms23158710 (PMC9369426; doi:10.3390/ijms23158710)
Supplement: Supplementary file 1 [file ijms-23-08710-s001.zip › ijms-1845998-supplementary.pdf]

Supplementary Materials

Enhancing the amyloid-β anti-aggregation properties of curcumin via arene-ruthenium(II) derivatization

Massimiliano Cuccioloni [1], Valentina Cecarini [1,] * Laura Bonfili [1], Riccardo Pettinari [2], Alessia Tombesi [3], Noemi Pagliaricci [2], Laura Petetta [3], Mauro Angeletti [1] and Anna Maria Eleuteri [1]

[1]School of Biosciences and Veterinary Medicine, University of Camerino - Via Gentile III da Varano, 62032 Camerino (MC) - Italy

[2]School of Pharmacy, University of Camerino - Via Sant'Agostino, 62032 Camerino (MC) - Italy

[3]School of Science and Technology, University of Camerino - Via Sant'Agostino, 62032 Camerino (MC) - Italy

Biosensor binding study – Amyloid-ligand interaction

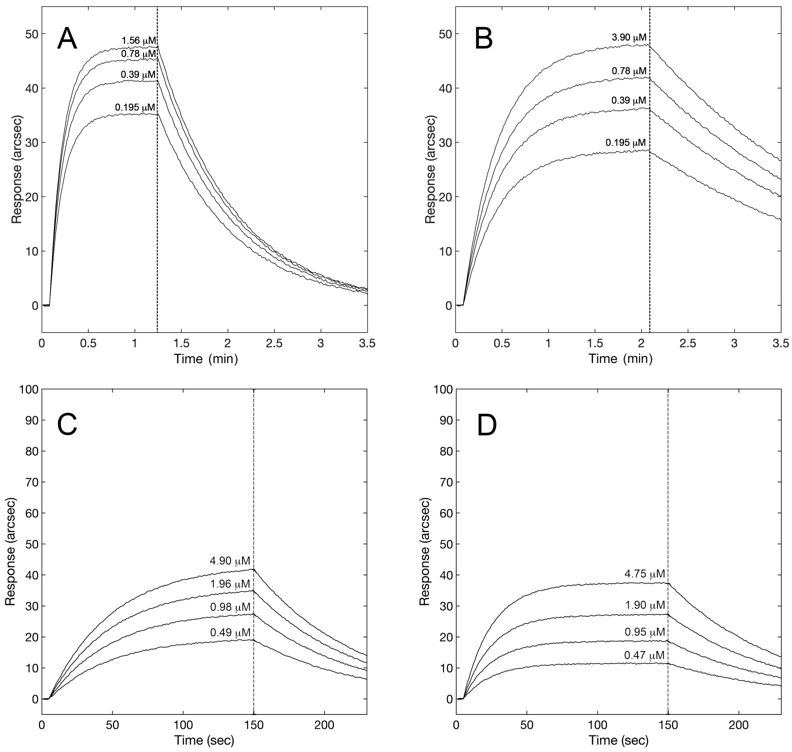

Figure S1. Representative sensor traces showing amyloid-ligand titration kinetics. Panels A and B compare the binding curves of curcumin and RuCurcumin to Aβ(1-40); panels C and D compare the binding curves of curcumin and RuCurcumin to Aβ(1-42).

ThT fluorescence – Amyloid aggregation kinetic parameters

Table S1. Effects on Aβ(1-40) fibrillogenesis. Best fitting parameters from fitting ThT fluorescence data to equation 1.

Fibrillogenesis Aβ(1-40)	*a*	*b*	*c*	*d*	*e*
**Aβ(1-40)**	$(2.8 \pm 2.2) \times 10^{-6}$	26.54 ± 3.45	487.7 ± 20.2	470.1 ± 11.6	2.183 ± 0.24
**Aβ(1-40)/Curcumin**	2.253 ± 1.54	20.23 ± 7.32	491.1 ± 37.2	124.2 ± 85.5	0.433 ± 0.38
**Aβ(1-40)/RuCurcumin**	1.384 ± 0.884	27.36 ± 5.76	492.2 ± 51.2	87.96 ± 9.06	0.289 ± 0.15

Table S2. Effects on Aβ(1-42) fibrillogenesis. Best fitting parameters from fitting ThT fluorescence data to equation 1.

Fibrillogenesis Aβ(1-42)	*a*	*b*	*c*	*d*	*e*
**Aβ(1-42)**	9.298 ± 7.343	14.4 ± 5.21	538.8 ± 83.2	739.8 ± 35.1	2042 ± 107
**Aβ(1-42)/Curcumin**	12.49 ± 2.85	9.15 ± 2.11	557.6 ± 36.4	370.8 ± 140.2	0.188 ± 0.14
**Aβ(1-42)/RuCurcumin**	$(8.8 \pm 6.57) \times 10^{-11}$	34.63 ± 2.95	527.5 ± 71.3	186.3 ± 11.5	0.4336 ± 0.32

Cell membrane permeability – Fluorescence anisotropy

Table S3. Rate constants for membrane entry (k_{in}) and release from membrane (k_{out}) of curcumin and RuCurcumin.

**Compound**	***k_{in} (s^{-1})***	***k_{out} (s^{-1})***
Curcumin	0.06 ± 0.03	0.005 ± 0.001
RuCurcumin	0.01 ± 0.003	0.008 ± 0.004

SEM analyses

Table S4. Mean diameters of amyloid fibrils.

	1-40 Fibril diameter (nm)	***1-42 Fibril diameter (nm)***
**Control**	99 ± 47	123 ± 63
**Curcumin**	73 ± 22	62 ± 9
**RuCurcumin**	50 ± 11	35 ± 8

Effect on cell viability

Table S5. Cytotoxicity (IC50, μM) of curcumin and RuCurcumin toward normal upon 4 h and 24 h exposure to SH-SY5Y

	nSH-SY5Y at 4h	nSH-SY5Y at 24h	SH-SY5Ywt at 4h	SH-SY5Ywt at 24h	SH-SY5Ymut at 4h	SH-SY5Ymut at 24h
**Curcumin**	>150	55.76 ± 16.1	>150	50.88 ± 17.4	>150	53.12± 13.2
**RuCurcumin**	>150	127.3 ± 10.1	>150	132.8 ± 7.7	>150	119.7 ± 9.7
